# Validation of the Spanish version of the Edinburgh feeding evaluation in dementia scale applied to institutionalized older persons with dementia: a study protocol

**DOI:** 10.1002/nop2.48

**Published:** 2016-04-06

**Authors:** Maria Carmen Saucedo Figueredo, Juan Carlos Morilla Herrera, Roberto Ramos Gil, Maria Nieves Arjona Gómez, Felicisima García Dillana, Javier Martínez Blanco, Jose Miguel Morales Asencio

**Affiliations:** ^1^ Los Boliches Health CentreCosta del Sol Primary Health Care DistrictMálagaSpain; ^2^ Nursing Home UnitMálaga‐Gualdalhorce Primary Health Care DistrictMálagaSpain; ^3^ Faculty of Health SciencesUniversity of MálagaMálagaSpain; ^4^ Nursing Home UnitCosta del Sol Primary Health Care DistrictMálagaSpain; ^5^ Department of Nursing and PodiatryFaculty of Health SciencesUniversity of MálagaMálagaSpain

**Keywords:** Aged, Dementia, Feeding behavior, Geriatric Assessment, Psychometrics

## Abstract

**Aim:**

The aim of this study was to obtain a Spanish version of the Edinburgh Feeding Evaluation in Dementia Scale version, to assess its reliability for use by medical staff and caregivers at residential care homes, to evaluate by confirmatory methods its construct validity. A further aim was to determine the criterion validity with respect to biochemical markers of malnutrition such as serum albumin, transferrin, cholesterol and lymphocytes, the body mass index and the mini nutritional assessment.

**Design:**

Clinimetric cross‐validation study.

**Methods:**

Institutionalized subjects with dementia will be observed while consuming meals and evaluated with the instrument independently by nurses and caregivers.

## Introduction

By 2050, people aged 60 years and over will account for 22% of the world's population (OMS, 2012). Spain will have the oldest population in the world after Japan (Clavé *et al*. 2007, Clavé 2012) and one in three Spaniards will be over 65 years old. Among those aged over 65 years, the fastest growing group will be those over 80 (INE, 2013).

Dementia is the third most common disease among older people, after cardiovascular and osteoarticular disease, with an incidence of 5‐10 cases per 1000 persons/year and this percentage rises with age, especially after the age of 85 (Sosa *et al*. 2005). Nutrition appears to be a predictor of satisfactory ageing and malnutrition continues to be a major geriatric syndrome and a risk indicator for morbidity and mortality (Ruiperez Cantera 2003). Among older persons, only 45% consume a healthy diet (Albert Cuñat *et al*. 2000). Even when they are healthy, older persons often have difficulty in feeding themselves, due to the natural consequences of the ageing process. Dysphagia is estimated to affect 7‐22% of the ‘healthy’ over 65s, rising to 25‐50% among those who also have dementia (Clavé *et al*. 2007, Palmer & Metheny 2008, Clavé 2012). Feeding difficulty is defined as any condition that may cause reduced food intake Watson, 1993a. It may appear in one or more of the following areas (Chang & Roberts 2011): initiating feeding independently, maintaining attention, taking food to the mouth and maintaining it there, chewing and swallowing. Dementia can cause feeding difficulties due to cognitive impairments as well as the physical disabilities that occur with the progression of the disease (Lin *et al*. 2010).

### Background

According to Durnbaugh *et al*. (1993), 70% of patients with advanced dementia have feeding difficulty. In general, it is accepted that dementia affects nutrition almost from the outset, producing anorexia, weight loss and eating/swallowing difficulties Gómez‐Busto *et al*. (2009). Proper nutrition is also affected by depression, which appears in 45% of cases (Morley & Kraenzle 1994). Malnutrition affects physical health and increases morbidity and mortality; consequently, the quality of life decreases (WHO, 2006, 2014, Zenon & Villalobos Silva 2012). The outcome may be even worse if the older person with dementia is institutionalized (Sandman *et al*. 1987, Watson 1997, García de Lorenzo y Mateos & Ruipérez Cantera 2007, Bermejo *et al*. 2014). It has been reported that among such institutionalized persons, 40% present dysphagia (García de Lorenzo y Mateos *et al*. 2012) and that more than half lose some ability to feed themselves (Leclerc *et al*. 2004). It is important to detect and identify the presence of feeding difficulty as soon as possible, however, over half of the feeding difficulties that affect older persons with dementia are due to factors unrelated to dementia itself, such as poor appetite, fatigue, depression, use of neuroleptics, oesophageal reflux or distraction environmental factors (Slaughter *et al*. 2011).

To identify feeding problems, some instruments have been proposed such as the Edinburgh Feeding Evaluation in Dementia Scale (EdFED) (Watson 1993a), the Feeding Behavior Inventory (Durnbaugh *et al*. 1993), the Aversive Feeding Behavior Inventory (Reyes Ortega 1996), the Eating Behavior Scale *(*Tully *et al*. 1998) and the Feeding Abilities Assessment (Leclerc *et al*. 2004). Of these, the EdFED is the most commonly used (Watson 1994, Watson & Deary 1994, Lin & Chang 2003, Aselage 2010, Aselage *et al*. 2011). This 11 items instrument informs not only of the presence of feeding difficulty but also of changes in the level of difficulty, thus guiding clinical intervention (Watson 1993b, 1997, Watson & Deary 1994) and the scale has also sensitivity to detect small changes in advanced dementia patients (Watson & Deary 1994, Watson *et al*. 2012). The first four items are related to patient assistance needs, six of them correspond to behaviours (initiating feeding independently, maintaining attention, taking food to the mouth and maintaining it there, chewing and swallowing) and the 11th to the intensity of help needed.

It has good internal consistency (Cronbach's alpha 0·87) (Watson 1996), reasonable interobserver reliability (*r* = 0·59) and good test‐retest reliability (*r* = 0·95) (Watson *et al*. 2001). Based on confirmatory factor analysis, two, three and four‐factor models have been constructed (Watson & Deary 1997), although these have not been tested in different contexts to determine their invariance. It has been adapted for use in the Chinese language (Lin & Chang 2003, Lin *et al*. 2008, Liu *et al*. 2014).

Data can be collected by direct observation of the patients as they eat and/or by questioning the formal caregivers or family members (Amella 2002, Chang & Lin 2005, Stockdell & Amella 2008). The pattern that is deduced is cumulative and hierarchic (Watson & Deary 1994, Watson *et al*. 2012).

In Spanish, available instruments applied to people with dementia are focused mainly on nutritional status, mostly applied in hospitals and they are not specifically designed for people with dementia. Examples include Subjective Global Assessment (Detsky *et al*. 1987), Nutritional Risk Screening (Kondrup *et al*. 2003), the Malnutrition Universal Screening Tool (Todorovic *et al*. 2003, Stratton *et al*. 2004, 2006) and the mini nutritional assessment (MNA) instrument (Guigoz *et al*. 1994).

Feeding difficulties in patients with dementia could lead to impairments on nutritional status and they may be associated with weight loss in long‐term care facilities. Due to eating behaviour disturbances, poor dietary intake and suboptimal diet can be found in people with dementia, so that it is necessary to evaluate whether eating difficulties could be related with changes in markers of nutritional status (Shatenstein *et al*. 2007, Sadamori *et al*. 2008).

At present, there are no instruments, validated in Spain, for use with institutionalized older persons to detect feeding difficulty, despite the increasing significance of this issue among a residential population that has tripled in recent years (INE, 2013). Moreover, no analysis has been carried out to explore the relation among feeding difficulties with EdFED and biochemical nutritional markers.

First, we aim to perform a cross‐cultural adaptation and validation of a Spanish version of the EdFED, among a residential population of older persons with dementia, for use not only by nurses but also by professional caregivers (staff in residential care homes). Second, we aim to determine the criterion validity of the scale with respect to biochemical markers such as serum albumin, transferrin, cholesterol and lymphocytes, the body mass index (BMI) and the MNA.

## The study

### Design

For the cross‐cultural adaptation and validation of the EdFED into Spanish language, we will develop a cross‐sectional psychometric validation study. Confirmatory methods will be used to evaluate the construct validity of the two, three and four‐factor structures of the EdFED and to determine the criterion validity regarding parameters such as: biochemical markers of malnutrition (albumin, cholesterol, transferrin and lymphocytes), BMI and MNA.

### Methods

The study will be conducted in residential care homes in the Costa del Sol healthcare district (Spain) in 2015. The study subjects will be aged over 65 years, institutionalized for at least 3 months and diagnosed with dementia. Exclusion criteria include terminal illness or other diseases that hinder feeding (stroke, Amyotrophic Lateral Sclerosis (ALS), motor neuron disease, maxillary fractures and paralysis.), the use of a gastrostomy tube, nasogastric tube, nasojejunal tube, enteral nutrition, refusal to participate in the study or the absence of consent by the legal guardians or reference relative/carers.

#### Sample

The sample size was calculated taking into account the capacity to detect older subjects with dementia and malnutrition (or the risk of it). Thus, assuming a prevalence of 21% of older people at risk of malnutrition (Camina Martín *et al*. 2012) among a total population of 1500 older institutionalized persons in the Costa del Sol Healthcare District, with an accuracy of 5% and an alpha of 0·05, 218 subjects would be required. With the same estimation parameters, assuming a prevalence of 10·8% of older persons with dementia and malnutrition (Dosil *et al*. 2013), 135 subjects would be required. To test the hypothesis for each of the two, three and four‐factor structural models, assuming a root mean square error of approximation (RMSEA) of 0·05, with an alpha value of 0·05 and a maximum of 66 d.f., a sample size of 175 subjects would be required. To meet all these sampling requirements and assuming a potential dropout rate of 15%, 251 subjects should be included. The sample size statistical calculations were carried out using Statistica 12 (StatSoft Inc., 2012) and Epidat 4·1 (Conselleria de Sanidade and Xunta de Galicia, 2014) software. The study will consist of the following phases.

#### Transcultural adaptation

With the permission of the original authors, the process of cultural adaptation will be addressed following the steps proposed by the International Test Commission for the adaptation of a scale (International Test Commission, 2005). The translation into Spanish will be performed by six independent translators, with Spanish as their native language, three of whom will be aware of the study goals and the others will not, to facilitate conceptual and literary translation simultaneously. The resulting texts will be evaluated by 20 healthcare professionals who routinely work with this type of patients and the best‐considered text will be chosen as the final Spanish version (SPV), following the recommendations from Merenda and Spielberger (2005).

It will be back‐translated into English by three translators, who have not participated in the first phase and working independently of each other. These translators will be bilingual, with English as their first language and will be unaware of the purpose of the study. Each of those three will give a version; their versions will be compared with each other and with the SPV to identify possible differences. The Spanish resulting version SPV will be evaluated by a group of 10 experts, including bilingual persons and professionals who routinely provide health care to persons with dementia and feeding difficulties (professional caregivers at residential care homes, general practitioners, community nurses and nutritionists). These persons will evaluate the semantic and cultural equivalence of the text and also the comprehensibility of the items and of the instructions for scoring the results. These evaluators will be different individuals to the first and they will award a score of 1 to each such item if the equivalence is semantic and cultural, 2 if it is only semantic and 3 if there is no equivalence. In addition, the committee (a group of experts) integrated by health care professionals with experience in the care of people with dementia will evaluate the conceptual relevance of each item, using Lynn's content validity index (Lynn 1986), on a four‐stage scale: 1: irrelevant, 2: somewhat relevant, 3: relevant, 4: highly relevant. The minimum ratio for content validity to be accepted will be 0·8. Finally, the items will be tested against the Flesch‐Szigriszt readability index (Szigriszt Pazos 1993).

Finally, a pilot test will be conducted with the resulting version to test its comprehensibility. In this pilot test, the instrument will be administered to 20 subjects in residential care homes by nurses and professional caregivers at these centres. Both type of professionals will be asked to report any difficulty in managing the instrument, or the understanding of any item.

#### Data collection

Before using EdFED to evaluate feeding difficulties, the persons who will administer the scale (nurses and caregivers) will be trained in its use. The following data will be obtained from the subjects: Barthel index, Pfeiffer Test, Global Deterioration Scale (GDS‐Fast), MNA, weight and height (when this is impossible, it will be estimated from the triceps skinfold), BMI and blood analysis results (albumin, transferrin, total lymphocytes and cholesterol). Observations will be made of the patient eating, either alone or helped by the caregiver, with an observation period of 10‐15 minutes per meal (Watson 1994). A nurse and a professional caregiver (observers) will make independent observations of the two meals (lunch and dinner) for each subject, to apply the EdFED scale.

### Analysis

Inter‐rater reliability (among nurses, professional caregivers and family caregivers) will be tested by the Pearson correlation coefficient and the intraclass correlation coefficient. To evaluate possible variations in patient's behaviour between meals, two different meals will be observed by the same evaluator and compared with these correlation coefficients. Internal consistency will be assessed by Cronbach's alpha. Construct validity will be determined by confirmatory factor analysis, to confirm the validity of the proposed two, three and four‐factor proposals (as reported elsewhere). The fit of the models will be evaluated according to the following indices: the penalizing function (χ^2^/gl), which is indicative of a good fit with values below 3; root mean square error of approximation (RMSEA) and 90% confidence intervals, taking the cutoff value of 0·05 as representing a good fit; the normed fit index (NFI), the comparative fit index (CFI) and the goodness of fit index (GFI), with a 0‐1 range and for which the minimum value reflecting good fit is taken as 0·90; and the standardised residual root mean square index, indicating a good fit with values below 0·08 (Hu & Bentler 1999). Criterion validity will be analysed, by reference to the values for albumin, transferrin, cholesterol, lymphocytes, BMI, MNA and weight of food left on the plate, by the Pearson correlation.

### Ethics

The study has been authorized by the Primary Healthcare Management District where it will be carried out on 17 February 2015. Permission from the original author of the instrument was obtained by mail on 19 November 19 2014. The study was also approved the Costa del Sol Hospital Research Ethics Committee on 1 December 2014. Informed consent will be requested of the guardian or responsible family member.

The standards of good clinical practice and the ethical principles for research on human beings, as stated in the Helsinki Declaration and its subsequent revisions, will be observed at all times.

## Discussion

As the disease progresses in older people with advanced dementia, feeding behaviours appear that promote nutritional deterioration. It is necessary to identify and remedy the eating problems early. The EdFED is not only capable of identifying these feeding difficulties but also provides guidance for potential interventions in clinical practice (Watson 1993b, 1997, Watson & Deary 1994).

An understanding of the characteristics and performance of the instrument, depending on the type of caregiver who uses it, is of particular importance because in many cases, it will not be administered by registered nurses, but by nursing assistants, who are among the most common caregivers in residential environments. The varying levels of autonomy and professional competence of nursing assistants could affect the reliability of the scale (Liu *et al*. 2011).

In most cases, the tools used to assess nutritional aspects of patients with dementia are generic and focus on assessing malnutrition or dysphagia, rather than eating difficulty. Thus, the approach taken is partial and possibly delayed (Detsky *et al*. 1987, Kondrup *et al*. 2003, Todorovic *et al*. 2003, Stratton *et al*. 2004, 2006).

In addition, evaluation of the convergent validity with biochemical markers of malnutrition will enable us to investigate the extent to which the values of the instrument reflect possible changes in levels of albumin, ferritin, cholesterol or lymphocytes, which among older people are more sensitive to nutritional fluctuations (Walrand *et al*. 2001). This is a question that to date has received very little research attention.

### Limitations

In patients with low levels of awareness or hyperactivity, the use of this instrument may be more problematic. Moreover, if different caregivers provide feeding assistance, this might affect the study results, since their interaction with the subjects or the type of assistance provided could affect the subjects' behaviour during the meal (Chang & Lin 2005, Ullrich & McCutcheon 2008, Lin *et al*. 2010, Aselage *et al*. 2011).

Finally, it will not be possible to evaluate the instrument's sensitivity to change with the proposed study design. This would require a longitudinal approach.

## Conclusions

The creation of an assessment instrument that has been rigorously cross‐culturally adapted and validated to assess feeding difficulties among institutionalized older persons with dementia will reveal its construct validity for use in a cultural environment other than that for which it was originally designed. We will also be able to test the reliability of the instrument when it is used by different types of caregivers, as well as its correlation with other biochemical markers of malnutrition. This adapted instrument will facilitate the assessment of feeding difficulty by nurses and nursing assistants at residential care homes and may improve the organizational culture with respect to the feeding of older persons with dementia, guiding the development of interventions for this problem.

## Author contributions

All authors have agreed on the final version and meet at least one of the following criteria [recommended by the ICMJE (http://www.icmje.org/recommendations/)]:

- substantial contributions to conception and design, acquisition of data, or analysis and interpretation of data;
- drafting the article or revising it critically for important intellectual content.
